# Wireless technology is an environmental stressor requiring new understanding and approaches in health care

**DOI:** 10.3389/fpubh.2022.986315

**Published:** 2022-12-20

**Authors:** Julie E. McCredden, Naomi Cook, Steven Weller, Victor Leach

**Affiliations:** ^1^Oceania Radiofrequency Scientific Advisory Association (ORSAA), Brisbane, QLD, Australia; ^2^Centre for Environmental and Population Health, School of Medicine and Dentistry, Griffith University, Brisbane, QLD, Australia

**Keywords:** environmental toxins, environmental health, environmental illness, electromagnetic hypersensitivity, wireless technology in health care

## Abstract

Electromagnetic signals from everyday wireless technologies are an ever-present environmental stressor, affecting biological systems. In this article, we substantiate this statement based on the weight of evidence from papers collated within the ORSAA database (ODEB), focusing on the biological and health effects of electromagnetic fields and radiation. More specifically, the experiments investigating exposures from real-world devices and the epidemiology studies examining the effects of living near mobile phone base stations were extracted from ODEB and the number of papers showing effects was compared with the number showing no effects. The results showed that two-thirds of the experimental and epidemiological papers found significant biological effects. The breadth of biological and health categories where effects have been found was subsequently explored, revealing hundreds of papers showing fundamental biological processes that are impacted, such as protein damage, biochemical changes and oxidative stress. This understanding is targeted toward health professionals and policy makers who have not been exposed to this issue during training. To inform this readership, some of the major biological effect categories and plausible mechanisms of action from the reviewed literature are described. Also presented are a set of best practice guidelines for treating patients affected by electromagnetic exposures and for using technology safely in health care settings. In conclusion, there is an extensive evidence base revealing that significant stress to human biological systems is being imposed by exposure to everyday wireless communication devices and supporting infrastructure. This evidence is compelling enough to warrant an update in medical education and practice.

## Introduction

Environmental illness often comes as a surprise to scientists and doctors alike. Environmental causes for human maladies are not always featured in formal training, yet they have accompanied many man-made innovations, from perfume and paint to petrol and plastics (1). It should not be surprising then that the modern world, saturated with technology, would impose effects on human biological systems, which are built from electrochemical processes. Electromagnetic fields and electromagnetic radiation, both natural and manmade, permeate the modern world. In particular, communications technology has become ubiquitous, with devices and transmitters placed in workplaces, homes, schools, hospitals and surrounding suburbs. The frequencies for relaying communications signals are collectively referred to as “radiofrequency” (RF). Examples of everyday technologies that use radiofrequencies include Wi-Fi routers, mobile phones, cordless phones, suburban towers, masts and panels on buildings (including hospitals), Bluetooth devices, smart meters, Fitbits, smart watches, baby monitors, game consoles and smart diapers (nappies).

The evidence base regarding the effects of ever-present electromagnetic pollution on health indicates that it acts like a stressor, placing an increasing burden on human biological systems (2, 3). However, while there have been some positive shifts in recent WHO Health topics, incorporating the effects of water and air pollution, endocrine disrupters, mercury and climate change, there has been very little focus on investigating electromagnetic pollution as an environmental stressor (4).

While much of the medical world remains ignorant regarding this environmental stressor, patients suffer (5). Such has been the clinical experience of one of the authors of this paper. People with hypersensitivity to electromagnetic fields may present to hospitals or clinics with an array of complaints that may or may not be based on their underlying condition, e.g., a bone fracture or a heart condition. While waiting, or during treatment, they may ask to be separated from mobile or cordless phones. Health care workers, not having heard of the condition of electromagnetic hypersensitivity or not having an understanding of the biological effects that are associated with electromagnetic fields, can find such requests strange or confusing and are unable to respond appropriately (6). This unmet need in care settings has motivated this paper, aimed at assisting the broader health profession with an understanding of how electromagnetic fields can affect human biology and providing guidance on how to respond to electrosensitive patients.

There also exists a level of ignorance surrounding this issue across the radiation protection profession. As a retired radiation protection practitioner, one of the authors of this paper has firsthand experience of how the busy daily working life in radiation protection involves a narrow focus on sources of ionizing radiation, with very little involvement, if any, on non-ionizing radiation devices that emit RF signals. Furthermore, if the need to investigate RF exposure arises, professionals seek advice from groups like the International Commission on Non-Ionizing Radiation Protection (ICNIRP), trusting that these bodies are honestly applying the ethical principles and risk reduction philosophies established by the International Commission on Radiation Protection (ICRP). The section on Public Safety Issues below discusses the unfortunate lack of precaution associated with RF technologies.

Despite the lack of official recognition, strong concerns for health and safety relating to radiofrequency emissions have recently entered the public arena. For example, in 2020, the Canton of Geneva placed a 3-year moratorium on fifth generation (5G) wireless technology (7) in response to public concerns and the lack of research into the effects of 5G on health and biodiversity. More recently, the US Court of Appeals for the D.C. Circuit (8) has ruled that the US Federal Communications Commission (FCC) has been negligent in its role as protector of the public's health over the last two decades by failing to consider the non-cancer evidence regarding adverse health effects and environmental effects of wireless technologies. Given this significant ruling, health care workers need to build an understanding of the RF exposure-induced health effects and their implications for medical practice.

### Evidence base

Health clinicians and policy makers must be assured that sound science is behind any claims that RF is an environmental stressor. The first section of this overview paper addresses this need, by summarizing the current scientific and medical evidence base that explores biological harm from everyday devices and wireless infrastructure in the built environment.

The scientific and medical evidence base that explores biological harm from low-level exposures to radiofrequency (i.e., non-thermal effects) was reviewed over a decade ago by a team of independent scientists and public health professionals who compiled *The BioInitiative Report* (9–11). This report summarized the evidence for an array of biological and health issues, including reduced fertility, neurological and behavioral effects, childhood leukemia, effects on gene and protein expression, and effects on immune function as well as cell stress responses. The 2020 *BioInitiative Report* update (11) comprises an extensive set of abstracts, tables, research summaries and the balance of evidence i.e., the number of studies showing effects vs. no effects. [Note: While there was some initial criticism of *The BioInitiative Report* as being biased and not peer-reviewed, many of the chapters were later published as peer-reviewed publications, e.g., (12), thereby laying these criticisms to rest].

The Oceania Radiofrequency Scientific Advisory Association (ORSAA) (13) has developed the world's largest categorized database of scientific studies on the biological and health effects of electromagnetic fields on humans, animals and the environment (14). The ORSAA Database of EMF Bioeffects (ODEB) (14) was first established using the entire research database of the Australian Radiation Protection and Nuclear Safety Agency (ARPANSA), and has been continually expanded, developed and refined ever since. ODEB currently comprises over 4,000 peer-reviewed publications (of which, over 2,400 are radiofrequency papers as of May 2022), including early military studies from the 70's, biophysics research from the 80's (before mobile phones) onwards, and a comprehensive collection of experimental and epidemiological research from both industry and independent scientists since 2012.

ODEB is continually being updated; i.e., two primary sources that were used to establish ODEB are now utilized on an ongoing basis for accessing candidate studies pertaining to ELF and RF frequencies. These are the US National Library of Medicine PubMed database and the ARPANSA technical series documentation, along with their EMR monthly literature surveys with reviews. Papers from the EMF-Portal of Rheinisch-Westfälische Technische Hochschule (RWTH) at Aachen University are also included if they pertain to EMF/EMR and health or bioeffect outcomes lower than the official ICNIRP thresholds (see below). This latter database contains many papers pertaining to EMR science that are not relevant to typical day-to-day public exposures, such as medical procedures and applications (ablation and diathermy) and electrical injuries, which are considered off-topic. Furthermore, many of the EMF-Portal papers describe results of exposures from power densities that create well-established heating effects that are protected against by current RF Guidelines and national RF exposure standards. ODEB, on the other hand, is more narrowly focused on studies using RF exposure levels sitting at approximately the ICNIRP exposure limit or below. These levels were chosen because this is where the crucial experiments have been performed to address the pertinent issues of bioeffects occurring at typical everyday public exposure levels and the subsequent appropriateness, or not, of current national RF standards. Articles are not selected for ODEB on the basis of positive bioeffect findings; i.e., there has been no “cherry-picking” of papers for inclusion. A comparison between the EMF-Portal and ODEB was carried out for relevant papers finding strong agreement on the number of studies (15) for the focus area.

ODEB is a true relational database with extensive search capabilities and is only limited by the categorization field set that is made available. This set is quite comprehensive in that ODEB is searchable by experimental category, biological endpoints, funding type, and many other variables^1^

Overall, we believe that the current evidence base regarding the effects of radiofrequency on biological processes is comprehensively represented by the structured collection of research papers in ODEB. The opinions given below are based on the weight of evidence emerging from ODEB and the papers from within this collection that describe effects.

The research papers in ODEB have been classified by ORSAA into major biological and health effects categories. The main categories discussed in the literature and used within ODEB are:

- DNA and cell damage in the brain, blood, body organs, immune and reproductive systems;
- Increased production of free radicals leading to a state of oxidative stress, and resulting in accumulated damage throughout the body;
- Neurodegeneration and blood-brain barrier breaches;
- Changes to neurotransmitter levels and signaling pathways in the brain;
- Damage to sperm and ovaries;
- Endocrine system effects;
- Damage to cellular systems and components such as mitochondria, mast cells and alterations to cellular signaling systems.

Damage to these processes underlies many health conditions.

Papers in ODEB are classified as an “Effect” paper if any one of the end-points reported in experiments within the paper is statistically significant (*p* < 0.05). Over two-thirds of the recent UHF (300 MHz-3 GHz) studies in ODEB contain significant effects of radiofrequency on biological systems or health, as described below.

## Evidence for biological effects of radiofrequency exposures

### Selection criteria

**Figure 1:**
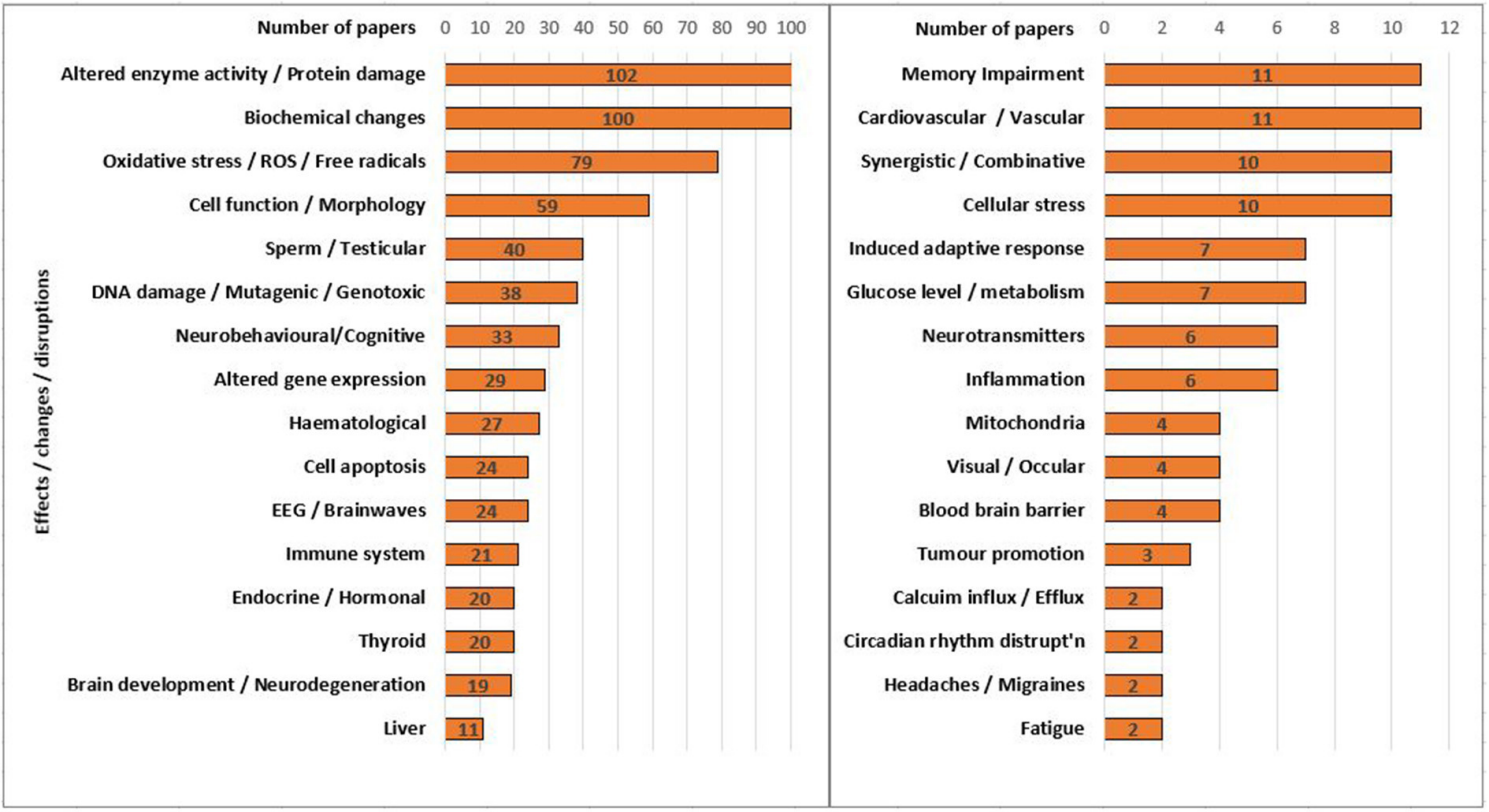
The number of selected experimental papers (using real-world signals) within the main bioeffects or health effects categories in ODEB.

This overview focuses on papers that use real-world exposure signals from everyday devices and communications infrastructure because our claim is that these technologies are an RF-based environmental stressor on biological processes and as a consequence, on health. Moreover, we claim these effects occur at and below the exposure thresholds set by the International Commission on Non-Ionizing Radiation Protection (ICNIRP; refer to section Public safety issues below). For this overview, papers were selected that were able to test our claims. Selection criteria included (1) relevance, (2) quality of reporting, and (3) quality of experimental design, as explained below. Note that this paper is not intended to be a systematic review; however, the structures contained in ODEB provide a helpful resource for such a review in the future. For example, the flow chart in Supplementary Data Sheet 3 summarizes the selection process used to extract the data used for Figure 1, using the layout of a Preferred Reporting Items for Systematic reviews and Meta-Analyses (PRISMA) flow chart.

Relevant for selection were those papers with stimulus signals in the radiofrequency range 300 kHz to 300 GHz and with exposure intensity levels below the ICNIRP thresholds. The subset of these relevant papers that were epidemiological studies (*n* = 251) were all accepted because, by their very nature, they investigate the effects of “real world” signals on residents living near mobile phone towers/base stations. However, there was also a large group of experimental papers that required further filtering. For these, quality in reporting was determined by the rejection of papers that, although relevant, gave a poor description of the signal type used; i.e., of the 1,343 relevant experimental papers in ODEB, 237 were rejected based on poor reporting, leaving 1,106 experimental papers remaining.

The quality of the experimental design was then determined based on whether the experiment used real-world signals instead of simulated signals. This selection criterion was deemed important (i) because the investigation focused on real-world exposures, and (2) because previous studies have noted that real-world signals (e.g., from mobile phones) are more likely to produce experimental biological interference effects than simulated laboratory signals that using synthesized, regular patterns (16). Even though simulated signals may be easier to control in experimental settings, they do not allow the experimenter to explore the essential factors that seem to cause stronger biological effects. This is possible because real-world devices emit constantly varying signals, to which human psychophysical systems appear to struggle to adapt, or because they contain pulses that elicit greater biological responses when compared to continuous waves of the same frequency (17). These pertinent factors need to be the focus of future research.

Indeed, the data from ODEB (see Table 1) corroborates the above research findings, by showing that the type of signal used: real or simulated, can affect study outcomes. Within the 1,106 relevant experimental papers selected from ODEB using the quality of reporting criteria above, there were proportionally more “Effect” outcomes when the experiments used real-world signals and proportionally more “No Effect” outcomes when simulated signals were used. This relationship between signal type and biological effect outcome was statistically significant (${\text{X}}_{1}^{2}$ = 21.2; *p* < 0.05), indicating that signal type needs to be clearly articulated in reporting because it can potentially bias outcomes. This result also supports our decision to investigate further only the experimental papers that used real-world signals. For these papers, shown in the final column of Table 1, there was a significantly higher proportion of papers showing effects (79.1%) than those reporting no effects (15.3%).

**Table 1:** Outcomes for selected experimental (*in vitro* and *in vivo*) studies.

**Study outcome**	**Relevant experimental papers in ODEB with wave form clearly described** ***N*** **(%)**	**Relevant papers with wave form clearly described, using simulated signals** ***N*** **(%)**	**Relevant papers with wave form clearly described, using real mobile phone or WiFi signals** ***N*** **(%)**
Effect	809 (73.1%)	221 (63.3%)	256 (79.1%)
No effect	229 (20.7%)	102 (29.2%)	49 (15.3%)
Uncertain effect	68 (6.2%)	26 (7.5%)	18 (5.6%)
Total	1,106	349	323

**Figure 2:**
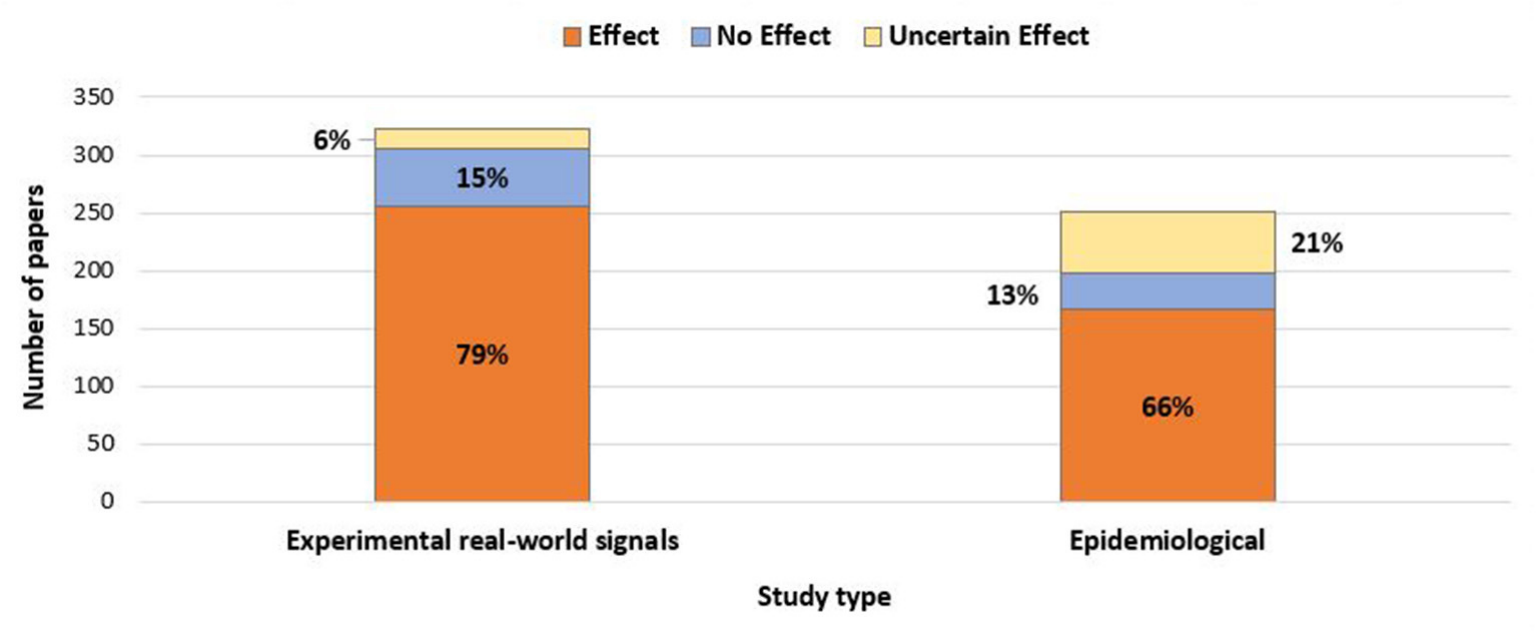
The relative numbers of selected experimental and epidemiological papers showing effects, no effects and uncertain effects.

Figure 2 illustrates the outcomes for the selected experimental papers compared to the epidemiology papers, showing that there was a similar pattern of more “Effect” papers existing for both study types. However, epidemiology studies have a larger proportion of “Uncertain Effects”. This is probably because epidemiology studies rely on finding a statistical association between increased exposures to base stations and higher numbers of affected people. Subsequently, the results are likely to be influenced by many potential confounders, co-causal factors (18), and sources of noise. It thus becomes more difficult to find strong effects from one variable amongst the many in such epidemiology studies. For example, because people today are surrounded by ELF and RF fields in everyday life i.e., personal wireless devices, it is difficult to isolate distance from a tower as a separate study variable that can indicate actual exposure levels or even find unexposed controls. Despite these potential weaknesses, epidemiology studies dominate RF research when it comes to exposure of human participants. Figure 2 shows that two-thirds of the relevant epidemiology papers selected from ODEB showed effects associated with increased exposures. Details of the ODEB search results used to construct Figure 2 are given in Supplementary Data Sheet 1.

### Sufficient evidence to motivate change

Results like those shown in Figure 2 provoke the question: “What constitutes enough evidence to support the claim that RF is an environmental stressor causing biological and health effects?” This issue of *sufficient evidence* has been explored in great detail in *Late Lessons from Early Warnings, Part E: Implications for science and governance*. In this report commissioned by the European Union, Gee (18) explains to all stakeholders that the strength of scientific evidence is not all or nothing; rather, it varies on a scale from *very weak* to *very strong*. That is, if the evidence is not yet conclusive, this does not mean there is no important evidence. There can still be *sufficient* evidence for governments and decision-makers to act so as to protect the health of humans, animals and plants from potential risks. The results shown in Table 1 and Figure 2 make the case for recognizing *sufficient* evidence exists and subsequently raising RF bioeffects in the environment as an important issue for decision-makers and providers of health policy, prevention and care.

This paper is aimed at those people who are responsible for setting health policy as well as those involved in health care. Given that the data above has established strong evidence, we now present in more detail the types of biological and health effects that have been shown to occur from real-world RF exposures.

## Types of effects observed

Figure 1 illustrates the biological effects categories underpinning the Experimental bar chart shown in Figure 2. The categories and counts in Figure 1 were taken from an ODEB summary page based on a search for the 323 selected experimental “Effect” papers [see Supplementary Data Sheet 3, part (iii)]. This selection is from papers that use real-world signals in their experimental design. For example, a search in ODEB for experimental papers that satisfied all of the selection criteria and found effects due to Oxidative Stress/ROS/Free radicals resulted in 79 papers, shown as one of the categories in Figure 1 (These Oxidative stress papers are also listed in detail within Supplementary Data Sheet 1).

The main narrative of this paper has been drawn from the above weight of evidence, that biological effects from low level everyday radiofrequency exposures exist. The sections below review papers that are illustrative of the main categories of effects found within ODEB; in particular, those with potential implications for human health. However, there are papers contained within ODEB that show no effects. These papers are not presented; rather, it is left to the reader to explore these papers using the online ODEB resource.

### Conditions arising from oxidative stress

Oxidative stress is now recognized as an underlying cause of many chronic diseases, such as cardiovascular disease and diabetes, Alzheimer's disease and depression. The Swiss expert research group BERENIS recently reviewed this topic and reported that most animal studies and more than half of the cell studies provided evidence of increased oxidative stress from electromagnetic fields, even in the low-dose range (19). Health conditions promoted by electromagnetic-induced oxidative stress include allergies and atopic dermatitis, autoimmune diseases such as diabetes, eye conditions, and fertility effects. Papers relating to all these topics can be found by searching ODEB online.

### Cancer

In 2011, The International Agency for Research on Cancer (IARC) classified radiofrequency as a Class 2B, Possible Carcinogen (20). More recently, the National Toxicology Program (NTP) results in the USA provided “clear” evidence linking radiofrequency exposure to cancer (21, 22). The $30M NTP study used exposure scenarios on rats that simulated human mobile phone exposure levels and higher. The results showed that male rats were more likely to develop malignant cardiac schwannomas than the unexposed control group. These rare nerve tumors have been previously linked to heavy cell phone use (23).

A similar animal experiment using lifetime exposures to low-intensity radiofrequencies equivalent to cell phone base station exposures was performed by the Ramazzini Institute. The same rare nerve tumors found in the NTP study were also found in the Ramazzini study. In light of these combined results, scientists globally have called for a WHO IARC upgrade of radiofrequency to a Class 1, “Known Carcinogen” category (24). The NTP study was used for a landmark legal case by the Turin Court of appeal (25), where the court confirmed that acoustic neuroma (vestibular schwannomas) can be caused by the occupational use of a mobile phone and that sufficient scientific evidence exists supporting this causal link.

### The vulnerable brain and children

In our technology-driven world, the human brain is constantly being subjected to everyday radiofrequency signals that cause structural and functional damage; e.g., to the hippocampus (26), the blood-brain barrier (27), mitochondrial energy metabolism (28) and neurotransmitters (29), which lead to negative consequences such as reduced spatial memory, unexplained headaches, reduced sleep performance, and neurological, cognitive and emotional disorders (29–32). Children's brains are especially vulnerable to damage and dysfunction because their skulls are thinner, and their brains absorb more radiation (33, 34). Children are now being exposed to radiofrequency from before conception. Based on a 10-year longitudinal study showing declines in an array of psychophysiological indicators, Grigoriev and Khorseva (35) concluded that children are a “group at risk” from mobile phone exposures. Children are now starting to appear in the long-term mobile phone user group (>10 years), which is the group most likely to be at risk of developing brain tumors (36, 37). Carpenter has warned that the cost of doing nothing may significantly harm a generation of young people who carry their mobile phones close to their bodies for many hours a day (38).

### Electromagnetic hypersensitivity: The canaries in the coal-mine

Some individuals suffer noticeable symptoms when exposed to radiofrequency or electromagnetic fields from telecommunications systems, electronic devices or electrical wiring. These symptoms are highly varied yet relate to classical symptoms of “microwave sickness” (39) which sufferers attribute to exposures to radiofrequency emitting devices or cell towers. These symptoms include headaches (not the typical headache), head pressure, chest pressure, dysesthesia (skin irritation) and paraesthesia (tingling, prickling, burning sensations), insomnia, concentration difficulties, tinnitus (ringing in the ears), memory issues, dizziness, heart problems such as arrhythmia/palpitations/tachycardia, anxiety, joint pain, chronic fatigue, muscle pain and dermatological effects such as rashes (40–42). “Electromagnetic hypersensitivity” (EHS) is the common term used to describe this condition. It is classified in the International Classification for Diseases, ICD-10, under category W90: *Adverse health effects of exposure to RF-EMR* (43). The WHO recognizes electromagnetic hypersensitivity as “idiopathic environmental intolerance” (44) but not the cause, and in Sweden it is recognized as a functional impairment (45).

Before mobile phones existed, Frey (46) found robust evidence that humans have a sensory system tuned to microwave frequencies. Moreover, these frequencies induced blood-brain barrier penetration and altered the brain's opiate-dopamine system, likely causing the headaches reported by Frey's research participants (47). Frey concluded that a person reporting headaches from mobile phone exposures might be a *canary in the coal mine warning of other biological effects* [47, p. 102].

More recently, medical researchers have found further evidence to establish electromagnetic hypersensitivity as a real condition:

- **Environmental factors implicated**: Electromagnetic hypersensitivity often occurs after prolonged exposures to electromagnetic fields at work or after medical examinations using X-rays or strong magnetic fields (48);
- **General sensitivity to toxins**: People with electromagnetic hypersensitivity have more frequent common colds, are more sensitive to chemicals, and are more likely to be affected by environmental factors such as car exhaust and dental amalgam (49);
- **Neurological damage:** Consistent evidence of physiological damage to nerves associated with using a mobile phone has been found by medical researchers (50, 51);
- **Brain changes:** People with electromagnetic hypersensitivity show different fMRI patterns (52);
- **Biomarkers:** Blood and saliva tests for diagnosing electromagnetic hypersensitivity are used by doctors aware of the condition, e.g., histamine levels are used to indicate inflammation, and serum malondialdehyde level is used to indicate oxidative stress from cell damage (53, 54);
- **Not psychosomatic:** A large proportion of people with electromagnetic hypersensitivity are not cognisant of any harm from radiofrequency prior to experiencing symptoms (40, 55). Thus, an “expectation of harm” i.e., nocebo effect cannot be used as the explanation for the condition.

Together, the above results provide converging evidence for the existence of human sensitivity to electromagnetic fields. EHS has recently been reframed as existing at the extreme end of a continuum whereby all humans have some level of electro-awareness and sensitivity but where individuals have varying abilities to repair damage from EMFs due to oxidative stress and other mechanisms (56). A recent review of EHS research based on the underlying biological mechanisms has noted the need for more relevant diagnostic tests for EHS (42). The lack of health policies for dealing with EHS has been found to be a global issue, prompting calls for the WHO to develop and promote health policy to assist EHS sufferers (57).

### 5G effects

Man-made radiofrequency is being added to dramatically with 5G signals, including thousands of small cell panels in every city, on street poles and apartment buildings in suburban areas, and close to hospitals and schools. The 5G signals differ from existing technologies because focused beams are used, and the new 5G plan is to use millimeter waves. Previously, millimeter waves were limited to police radar, military radar, and non-lethal weapons for crowd dispersal and airport scanners, which are not considered to be dangerous by authorities due to the short exposure times of use. However, current telecommunications systems emit constantly (24/7). The long-term effects on human health from beamed, pulsed millimeter waves have not been sufficiently studied, so that recent reviews have been unable to draw any strong conclusions (58) or have stated *no confirmed evidence* [(59), p. 601] and *little consistent evidence* [(60), p. 613]. Note that these statements do not mean *evidence of no harm* and must not be misconstrued that way (18). Furthermore, the review process itself can be biased or incomplete, as noted in (61).

Without adequate research, it is hard to formulate public policy, yet the 5G rollout is advancing nevertheless. Public regulatory bodies have attempted to reassure medical practitioners and the general public by advising that 5G millimeter waves will “only enter into the outer layers of the skin.” Such statements ignore the skin's critical biological functions, such as its role in the immune system, waste management and its rich innervation. The sweat duct ends within the epidermis have a helical shape, enabling them to act as antennas, drawing radiofrequency signals into the body (62, 63). Furthermore, the rapid, pulsed, narrow beams comprising 5G signals may cause intense hot spots, creating permanent skin and tissue damage (64). A recently declassified Russian study found an *unfavorable influence of millimeter radio-waves on the organism* [(65), p. 60] such as bunched and damaged nerves in the skin and surface layers, changes to protein and carbohydrate metabolism, and disturbances of the immune and blood systems. Bioactive and possibly dangerous exposures to 5G millimeter waves may create a cascade of biological events with unknown consequences (48). At the same time, Russian research has also shown that millimeter waves in specific frequency ranges given in very short doses (e.g., 15 min) can have therapeutic effects that act on the body via the skin (66, 67). Overall, there is inadequate research on the impact of 5G waves on the skin (68), and further investigations are paramount given the current rollout of these technologies and the non-consensual nature of most exposures.

One way to put this situation into context for health care professionals is to compare it with the concept of *polypharmacy*. When certain drugs are combined, sometimes no studies exist to demonstrate safety. In the case of polypharmacy, nurses are aware that there are risks involved in adding even just a few new drugs to a patient's regime, and that close monitoring for effects is crucial in such cases. Similarly, 5G is being added to the current 2G, 3G and 4G mixture of radiofrequencies. However, there has been no biological safety testing and no corresponding public health warnings about the analogous “polyfrequency” effects on biological systems occurring from multiple exposures to different types of radiofrequencies, including millimeter waves. The unknown effects of such layering of radiofrequencies are flagged in the guidelines of the International Commission on Non-Ionizing Radiation Protection (69). Despite this caution, no tests have been conducted for such potential additive effects, and the 5G rollout is advancing, unhindered by the current lack of research.

#### Implications for science and medicine

The above findings have far-reaching implications. From a postpositivist perspective (70) a scientific truth is being established *via* converging evidence from many sources and the rejection of alternative explanations. The fact being pointed out is that human systems interact with electromagnetic fields even at low power levels, which challenges the current understanding of human perceptual and signaling systems, and warrants further investigation (71). From a medical perspective, these results may give some clues about the rising levels in technology dominant countries of serious health conditions such as cancer, Alzheimer's disease and other forms of dementia, as well as illnesses in young people that are on the increase, such as depression, hyperactivity, Type II diabetes, hypertension and psychoses.

## Medicine and the lens of biophysics

Health education involves the study of human biology, physiology, biochemistry and anatomy. However, understanding how electromagnetic fields can harm human systems requires an understanding of biophysics i.e., how human biology responds to physical forces. Biophysics explains the electrically sensitive nature of the human body both at the basic level i.e., the role of electrical signaling in the heart, the brain, the nervous system, and also at a cellular level, the voltage-gated channels that enable cells to function and respond to the extracellular environment. The sections below briefly introduce the biophysics of electromagnetic radiation, describing some of the suggested mechanisms of action.

### Mechanisms of biological interaction

The literature shows that the effects of radiofrequency are dependent on various wave characteristics such as the frequency of the carrier wave, the frequency of the modulating wave (which rides on top of the carrier wave so as to define the information being carried; e.g., the text message being sent from a mobile phone), the intensity of the wave and whether the wave is pulsed or continuous (72). Therefore, to understand how electromagnetic fields interact with human biology, there is a complex puzzle to be solved with many dimensions to be considered. While the mechanisms are not yet fully understood, several plausible mechanisms have been postulated, as described below.

### Many charges vibrating coherently

Making up the biological system of the body are building blocks such as atoms, molecules and crystals. These small components of life all contain many charged components, which vibrate at various frequencies. Synchronized vibrations between large numbers of charged components can create very large electromagnetic forces that cause the molecules and other biological components to resonate with one another at specific frequencies (73, 74). This phenomenon, called “coherence,” may be fundamental to determining which interactions occur between biological components (e.g., between molecules) and even the shapes of various tissues. Fröhlich (73) warned that because the membranes of cells vibrate at millimeter wave frequencies, they would likely be affected by microwaves oscillating at the same frequency. Fröhlich thus predicted that 5G frequencies would disrupt cell membrane functioning.

### Inappropriate movement of Calcium ions

An oscillating electric field can cause inappropriate movement of Calcium ions across cell membranes (73, 74). One example is the inappropriate opening of the voltage-gated calcium channels in cell membranes. These are protein “gates” which sit on the external plasma membranes of cells, opening and closing when they detect a specific change in voltage in order to allow Ca++ ions to pass across. It has been shown theoretically that the oscillating electric and magnetic fields of a radiofrequency wave can cause the free calcium ions to vibrate with enough energy that they signal a “false” change in voltage to the gate proteins. This results in the inappropriate opening of the gates and an influx of Ca++ ions through the channels and into the cell (75, 76). Calcium influx due to the opening of the calcium gated channels can then result in a myriad of adverse intracellular activities, including nitrosative and oxidative stress, leading to downstream health effects (77, 78).

### Disruptive movement of charges

The disruptive movement of charges created by the vibrational frequency of a radiofrequency wave can move other charged ionic molecules in unexpected ways. One result is the redistribution of charges in protein molecules, leading to changes in protein structure and subsequent pathologies (79). Other outcomes include damage to cells, mitochondria, cellular stress, damage to proteins and DNA (80) as well as incorrect signaling between cellular and neural systems. This can further result in oxidative stress and inflammation, resulting from long-term exposures to disruptive radiofrequency forces, cellular repair mechanisms struggle to keep up with the damage from oxidative stress, resulting in DNA mis-repair and cancer (81) and cardiovascular disease (82).

Further understanding of possible mechanisms of action between electromagnetic fields and biological systems has been explored in more depth elsewhere (83–85). Main themes and research needs were also presented in 2017 at a European joint biomedical and engineering conference (86).

Currently, the above mechanisms are not fully understood and unfortunately used by some as a reason to downplay observed changes. In science, as well as in clinical practice, lack of understanding of the mechanisms of action does not detract from the observed data; e.g., aspirin was used as an anti-inflammatory for over 70 years before its mechanism of action was understood, and the inflammatory and immune suppressant effects of cigarette smoking are still being explored.

## Public safety issues

The above evidence basis for the biological effects of microwave radiation has far-reaching implications for public health policy. To date, these implications have not been reflected in the guidelines of international regulatory bodies such as the International Commission on Non-Ionizing Radiation Protection (ICNIRP) that governments look to for guidance on these matters.

### Official limits focus on thermal mechanisms only

One well-known mechanism by which radiofrequency wavelengths can damage living tissue is heating, a process well-understood by engineers and physicists. Unfortunately, damage from heating as the only mechanism of harm is assumed and used as the basis for the ICNIRP safety guidelines, which specify power density thresholds for radiofrequency exposures beyond which the public must not be exposed. Industry must comply with these limits to prevent short-term heating effects from medical devices, smart devices and wireless technologies. However, the types of biological harm from radiofrequency described in the sections above are not necessarily due to heating yet may lead to short- and long-term harm to the population. The guidelines do not protect against these biological effects because such effects occur at exposures with much lower power densities than ICNIRP guidelines permit, which set the “safety” thresholds at a power density of 10,000 milliwatts/m^2^ (69) and an average whole-body heat absorption (SAR) of 0.08 Watts/kilogram. At much lower power densities, Frey found the human heart is sensitive to pulsed signals (at 3 microwatts/cm^2^; i.e., 30 milliwatts/m^2^) (87), and Salford found dark neurons in the brains of rats (at 240 and 2,400 milliwatts/m^2^). A recent review of over 100 studies using very low intensity exposures has revealed that the median SAR at which effects occur is 0.0165 W/kg (3). These works, showing significant effects at very small doses, demonstrate that there is no clear linear dose-response relationship (where greater the RF power absorption, the greater the adverse effect). The invalid assumption of a linear-dose relationship is embodied within the ICNIRP guidelines. What is more true is that exposure dose is a product of both exposure intensity and time of exposure (3); however, the ICNIRP guidelines do not cater for exposures longer than 30 min. Moreover, the guidelines do not consider modulation and pulsing, which may be the more bioactive components of wireless signals (17). Altogether, these results position the ICNIRP guidelines as invalid and irrelevant to real-world exposure scenarios.

### Lack of protection for vulnerable populations

The most recent ICNIRP guidelines (69) do not have a stricter category for children, babies, fetuses, sperm or ovaries, thereby treating them all the same as adults; i.e., as “members of the public.” Exposures to members of the public are uncontrolled exposures and should protect against plausible risk, as specified by the International Commission on Radiation Protection (ICRP) (88). ICNIRP radiation protection philosophy does not align with ICRP best practices and does not provide protection or “opt-out” rights for people who do not want to be exposed, such as those with microwave sickness or electromagnetic hypersensitivity. This situation is also in contrast with the *Russian Federal Service for Surveillance on Consumer Rights Protection and Human Wellbeing (Rospotrebnadzor)*, which has heeded their scientists' warnings that children are in the “at risk” group (89) and has subsequently issued guidelines for schools and parents on the safe use of mobile phones. In addition, the governments of Cyprus, France and Israel have banned Wi-Fi in nurseries and schools.

Around the world, RF protection is determined at the national level, where exposure guidelines and limits for most countries are based on values derived by ICNIRP. However, there are ongoing debates about the adequacy of these guidelines, raising questions about whether they are sufficiently protective (90). There are also questions from researchers outside of WHO and ICNIRP, about the independence of ICNIRP from industry, and freedom of independent thought within ICNIRP (91). A recent review has shown that in the majority, ICNIRP affiliates belong to a small group of 17 self-referencing authors (92).

In the case of radiofrequency exposures, poor guidance and lack of transparency has meant that neither medical professionals nor the public has been adequately advised about potential harm. Consequently, patients may only realize their brain tumor is linked to their mobile phone usage when it is far too late, as was the case for the late Robert Kane, a previous telecommunications engineer and employee of Motorola (93). The situation is similar to the levels of medical and public awareness in the middle of the last century regarding the link between smoking and lung cancer.

### Invoking a precautionary approach

Globally, government regulators of medical services such as X-ray units take a precautionary approach to managing low-dose ionizing radiation, even though the long-term biological effects of very low doses of X-rays are considered uncertain. Precaution means that action needs to be taken to reduce risks, and the decision making behind these actions needs to be justified, open and transparent (94). The same philosophy must prevail with non-ionizing radiofrequency radiation in that the Precautionary Principle should be invoked in hospital settings, schools, workplaces, public places and in the home (95–97). Several countries have adopted a precautionary stance, such as China, India, Poland, Russia, Italy and Switzerland, regions of Belgium and cities such as Paris. Unfortunately, in most other countries, the current ICNIRP guidelines have been wholly adopted by government radiation protection agencies as standard without due consideration for risk assessment and protection (98).

## Practical applications for health professionals

The above overview shows how radiofrequency signals comprise an ever-present environmental stressor that may contribute to the significant increases in chronic illnesses and mental health issues observed globally. A list of suggestions surrounding best practices with issues related to radiofrequency exposures are offered below to assist health professionals in making the healthcare environment safer and to equip them to advocate for the health, safety and rights of their patients, e.g., as is articulated in the Code of Ethics for Nurses, Provisions 3, 5, 6–9 (99). Steps that health professionals can take include:

1. **Responding appropriately to patients with electromagnetic hypersensitivity**: When presenting to hospitals or clinics, EHS patients may ask for tablets and laptops or office Wi-Fi to be disabled (Airplane Mode on, Location Services off). They may also request for distancing from cordless phones, which can affect the heart (100). The uninformed health care worker may be confused and no know how to respond. To provide guidance, the Austrian Medical Association has written a set of Guidelines (6), which include recording patient history, examination, measurement, prevention or reduction of exposure, diagnosis and treatment. Prevention includes removing sources of radiofrequency by switching off, unplugging, shielding, distancing, or using wired connections as an alternative.
2. **Recording cases with links between symptoms and EMR exposures**: Peel (101) has suggested that professionals' experiences can help identify possible sources of uncertainty and evaluate the health risks due to various environments. Therefore, if health care workers record cases where they observe patients who seem to be affected by radiofrequency, this will provide data for positive change in the future.
3. **Being mindful of radiofrequency** as a possible contributor to illnesses when patients present. This includes developing an awareness of the radiofrequency emitting devices in the environment that may be causing distress to some patients (6).
4. **Self-education**: Healthcare workers typically strive to stay up to date with the current medical evidence base. Accordingly, they can investigate the effects of radiofrequency on the health of their patients and the general public. The references in this report provide a good grounding and helpful sites for medical professionals. For example, the Physicians' Health Initiative for Radiation and Environment in the UK provides practical “how-to”' advice (102), and has recently issued a consensus statement (103) regarding the health effects of radiofrequency exposures. The Environmental Health Trust^2^ provides short videos and modules explaining the primary research and applications, such as the effects of cell phones on the body (104).
5. **Giving guidance to patients:** Healthcare workers are in a position to advise on healthy lifestyles, particularly those working in Primary Care. In these settings, they can speak one-on-one with patients about the risks associated with mobile phone use and show them how to use their phones more safely. The existing online resources (6, 102, 104) can be used to devise educational leaflets for placement in clinics and waiting rooms.
6. **Broader considerations for health policy:** While individuals can change their practices, systemic approaches to this emerging health issue are also required, such as:
  - **Institutional education**: Doctors and other medical professionals, particularly those in psychiatry and psychology, need to understand how biological harm can occur from exposure to physical entities in the built environment and how the downstream effects may contribute to ailments in their patients. At the same time, courses that provide general training on epidemiological and toxicological aspects of environmental health (105) also need to include the biological and health effects of electromagnetic fields. A 21st-century tertiary curriculum incorporating biophysics needs to be designed for relevant graduate courses and modules for continuing professional development.
  - **Precaution in health care delivery:** Those responsible for the design and delivery of healthcare need to consider electromagnetic fields in the environment. In face-to-face settings, this means precaution in placing electromagnetic equipment and radiofrequency devices in waiting rooms and treatment areas.
  - **Policy needed for eHealth:** Internet-based health services bring many advantages; however, the technology needs to be used wisely. Because the health effects of EMFs are unknown to most practitioners and policy makers, eHealth practices are currently running ahead of the science and potentially causing harm. Patients and health professionals need to be aware of the potential adverse combinative effects that exposure to wireless devices may have on health conditions; e.g., using a wireless modem connection to consult a practitioner, a smartwatch to report a heart rate (106) or sending heart data wirelessly from inside an ambulance carrying a heart attack patient may all exacerbate heart injury (82). Wired solutions need to be put in place for all eHealth services where possible, and policy makers need to lobby research institutions to find appropriate eHealth solutions that first do no harm.

## Conclusion

Man-made radiofrequency signals from everyday devices and communications technology infrastructure constitute an environmental stressor, well-documented as creating various adverse biological effects. Plausible mechanisms in which harm can occur initially on a cellular level have been proposed, and these mechanisms are known to have subsequent downstream health effects. The application of the ICRP radiation protection philosophy and framework for the protection of members of the public is over 90 years in the making and is absent in setting exposure limits for this form of (wireless) radiation. The extensive evidence base is compelling enough to call for an update in medical education and practice. Out of care for their patients, healthcare workers may develop their understanding using the practical methods introduced in this discussion paper. Furthermore, modern institutional practices need to be reviewed to ensure that any harm from electromagnetic fields is reduced as much as reasonably possible while still providing optimal health care.

## Author contributions

All authors listed have made a substantial, direct, and intellectual contribution to the work and approved it for publication.

## Footnotes

^1^ODEB is a free online research tool (https://a037613.fmphost.com/fmi/webd/Research_Review_V4). To assist users to best use this database, a webinar has been created (https://www.orsaa.org/orsaa-database-training-webinar.html).

^2^ https://ehtrust.org

## References

[1] Harremoës P Gee D MacGarvin M Stirling A Keys J Wynne B. (Eds). Late Lessons From Early Warnings: the Precautionary Principle 1896–2000. Luxembourg: Office for Official Publications of the European Communities (2001). 10.4324/9781315071985

[2] Carpenter DO. Human disease resulting from exposure to electromagnetic fields1. Rev Environ Health. (2013) 28:159–72. 10.1515/reveh-2013-0016 24280284

[3] Lai H Levitt BB. The roles of intensity, exposure duration, and modulation on the biological effects of radiofrequency radiation and exposure guidelines. Electromagn Biol Med. (2022) 41:230–55. 10.1080/15368378.2022.2065683 35438055

[4] World Health Organization. Improving Environment and Health in Europe: How Far Have We Gotten? 2015, WHO Regional Office for Europe: Copenhagen. Available online at: https://www.euro.who.int/__data/assets/pdf_file/0018/276102/Improving-environment-health-europe-en.pdf (accessed October 28, 2022).

[5] Hardell L Carlberg M. Lost opportunities for cancer prevention: historical evidence on early warnings with emphasis on radiofrequency radiation. Rev Environ Health. (2021) 36:585–97. 10.1515/reveh-2020-0168 33594846

[6] Austrian Medical Association. Guideline of the Austrian Medical Association for the Diagnosis and Treatment of EMF Related Health Problems and Illnesses (EMF Syndrome). Vienna (2012). Available online at: https://www.avaate.org/spip.php?article2279 (accessed October 28, 2022).

[7] Home Radio Television Switzerland. Moratoire de trois ans sur la 4G+ et la 5G à Genéve. Available online at: https://www.rts.ch/info/regions/geneve/11125794-moratoire-de-trois-ans-sur-la-4g-et-la-5g-a-geneve.html (accessed October 28, 2022).

[8] United States Court of Appeals for the District of Columbia Circuit. Environmental Health Trust et al. vs Federal Communications Commission and United States of America, Ruling 20-1025, consolidated with 20-1138, On Petitions for Review of an Order of the Federal Communications Commission. (2021). Available online at: https://www.cadc.uscourts.gov/internet/opinions.nsf/FB976465BF00F8BD85258730004EFDF7/$file/20-1025-1910111.pdf (accessed October 28, 2022).

[9] BioInitiative Working Group. BioInitiative Report: A Rationale for a Biologically-Based Public Exposure Standard for Electromagnetic Fields (ELF RF) Sage C, Carpenter DO, editors (2007). Available online at: https://ccst.us/wp-content/uploads/Bioinitiative_report.pdf (accessed October 28, 2022).

[10] BioInitiative Working Group. Bioinitiative Report: A Rationale for a Biologically-Based Public Exposure Standard for Electromagnetic Radiation Sage C, Carpenter DO, editors (2012). Available online at: https://bioinitiative.org (accessed October 28, 2022).

[11] BioInitiative Working Group. Bioinitiative Report: 2020 Updated Research Summaries, Sage C Carpenter DO, editors (2020). Available online at: https://bioinitiative.org/research-summaries/ (accessed October 28, 2022).

[12] Blank M. Preface, Pathophysiology. Elsevier (2009). p. 67–9. Available online at: https://mreengenharia.com.br/pathfisology/Pathophysiology_2009_Preface.pdf (accessed November 29, 2022).

[13] Oceania Radiofrequency Scientific Advisory Association. (2015). Available online at: https://www.orsaa.org/ (accessed October 28, 2022).

[14] Leach V Weller S Redmayne M. A novel database of bio-effects from non- ionizing radiation. Rev Environ Health. (2018) 33:273–80. 10.1515/reveh-2018-0017 29874195

[15] Leach V Weller S Redmayne M. Authors' Reply to Drießen's Letter to the Editor on “A novel database of bio-effects from non-ionizing radiation. Rev Environ Health. (2019) 34:101–3. 10.1515/reveh-2018-0077 30710488

[16] Panagopoulos DJ. Comparing DNA damage induced by mobile telephony and other types of man-made electromagnetic fields. Mutation Res. (2019) 781:53–62. 10.1016/j.mrrev.2019.03.003 31416578

[17] Panagopoulos DJ Johansson O Carlo GL. Real versus simulated mobile phone exposures in experimental studies. Biomed Res Int. (2015) 2015:607053. 10.1155/2015/607053 26346766 PMC4539441

[18] Gee D. More or Less Precaution? in Late Lessons from Early Warnings: Science, Precaution, Innovation. EEA Report Vol. 2013 No. 1, Gee D, editors. European Environment Agency: Luxembourg (2013). p. 675–701. Available online at: https://www.eea.europa.eu/publications/late-lessons-2 (accessed October 28, 2022).

[19] BERENIS. Is there evidence for oxidative stress caused by electromagnetic fields?, in A Summary of Relevant Observations in Experimental Animal and Cell Experiments Related to Health Effects in the Last Ten Years, Mevissen M and Schürmann D, editors. Department of Epidemiology and Public Health Environmental Exposures and Health Unit: Basel, Switzerland (2021). Available online at: https://ehtrust.org/wp-content/uploads/Newsletter-BERENIS-Special-Issue-January-2021-1.pdf (accessed October 29, 2022).

[20] International Agency for Research on Cancer. IARC Classifies Radiofrequency Electromagnetic Fields as Possibly Carcinogenic to Humans. Press release (2011). Available online at: https://www.saferemr.com/2019/ (accessed on 29 October 29, 2022).

[21] Wyde M Cesta M Blystone C Elmore S Foster P Hooth M. Report of partial findings from the national toxicology program carcinogenesis studies of cell phone radiofrequency radiation in HSD: Sprague Dawley^®^ SD rats (whole body exposures). bioRxiv. (2018). 10.1101/055699

[22] Melnick RL. Commentary on the utility of the national toxicology program study on cell phone radiofrequency radiation data for assessing human health risks despite unfounded criticisms aimed at minimizing the findings of adverse health effects. Environ Res. (2019) 168:1–6. 10.1016/j.envres.2018.09.010 30243215

[23] Hardell L Carlberg M Söderqvist F Mild KH. Pooled analysis of case-control studies on acoustic neuroma diagnosed 1997-2003 and 2007-2009 and use of mobile and cordless phones. Int J Oncol. (2013) 43:1036–44. 10.3892/ijo.2013.2025 23877578 PMC3829779

[24] Hardell L Carlberg M. Comments on the US national toxicology program technical reports on toxicology and carcinogenesis study in rats exposed to whole- body radiofrequency radiation at 900 MHz and in mice exposed to whole-body radiofrequency radiation at 1,900 MHz. Int J Oncol. (2019) 54:111–27. 10.3892/ijo.2018.4606 30365129 PMC6254861

[25] Phonegate. The Court of Appeal of Turin Confirms the link Between a Head Tumour and Mobile Phone Use. (2020). Available online at: https://www.phonegatealert.org/en/the-court-of-appeal-of-turin-confirms-the-link-between-a-head-tumour-and-mobile-phone-use/ (accessed October 29, 2022).

[26] Li Z. Relationship between cognition function and hippocampus structure after long-term microwave exposure. Biomed Environ Sci. (2012) 25:182–8. 10.3967/0895-3988.2012.02.009 22998825

[27] Salford LG Brun AE Eberhardt JL Malmgren L Persson BR. Nerve cell damage in mammalian brain after exposure to microwaves from GSM mobile phones. Environ Health Perspect. (2003) 111:881–3. 10.1289/ehp.6039 12782486 PMC1241519

[28] Hao YH Zhao L Peng R-Y. Effects of microwave radiation on brain energy metabolism and related mechanisms. Mil Med Res. (2015) 2:1–8. 10.1186/s40779-015-0033-6 26000171 PMC4440565

[29] Hu C Zuo H Li Y. Effects of radiofrequency electromagnetic radiation on neurotransmitters in the brain. Front Public Health. (2021) 9:1–15. 10.3389/fpubh.2021.691880 34485223 PMC8415840

[30] Foerster M Thielens A Joseph W Eeftens M Röösli M. A prospective cohort study of adolescents' memory performance and individual brain dose of microwave radiation from wireless communication. Environ Health Perspect. (2018) 126:077007. 10.1289/EHP2427 30044230 PMC6108834

[31] Wang J. Mobile phone use and the risk of headache: a systematic review and meta-analysis of cross-sectional studies. Sci Rep. (2017) 7:1–7. 10.1038/s41598-017-12802-9 28974725 PMC5626766

[32] Lustenberger C. Stimulation of the brain with radiofrequency electromagnetic field pulses affects sleep-dependent performance improvement. Brain Stimul. (2013) 6:805–11. 10.1016/j.brs.2013.01.017 23482083

[33] Morgan LL Kesari S Davis DL. Why children absorb more microwave radiation than adults: the consequences. J Microsc Ultrastruct. (2014) 2:197–204. 10.1016/j.jmau.2014.06.005

[34] Gandhi OP Morgan LL de Salles AA Han YY Herberman RB Davis DL. Exposure limits: the underestimation of absorbed cell phone radiation, especially in children. Electromagn Biol Med. (2012) 31:34–51. 10.3109/15368378.2011.622827 21999884

[35] Grigoriev YG Khorseva NI. A longitudinal study of psychophysiological indicators in pupils users of mobile communications in Russia (2006-2017): children are in the group of risk. In:Markov M, editor. Boca Raton, FL: Mobile Communications and Public Health CRC Press (2018). p. 237–52.

[36] Hardell L Carlberg M Söderqvist F Mild KH Morgan LL. Long-term use of cellular phones and brain tumours: increased risk associated with use for? 10 years. Occup Environ Med. (2007) 64:626–32. 10.1136/oem.2006.029751 17409179 PMC2092574

[37] Yang M Guo W Yang C Tang J Huang Q Feng S. Mobile phone use and glioma risk: a systematic review and meta- analysis. PLoS ONE. (2017) 12:e0175136. 10.1371/journal.pone.0175136 28472042 PMC5417432

[38] Carpenter DO. Electromagnetic fields and cancer: the cost of doing nothing. Rev Environ Health. (2010) 25:75–80. 10.1515/REVEH.2010.25.1.75 20429163

[39] McRee DI. Soviet and Eastern European research on biological effects of microwave radiation. Proc IEEE. (1980) 68:84–91. Available online at: https://avaate.org/IMG/pdf/mcree80_rev_soviet.pdf (accessed November 26,2022).28846587

[40] Havas M. Electrosmog and electrosensitivity: what doctors need to know to help their patients heal. In:Klatx R Goldman R, editors. Anti-Aging Therapeutics Volume XV. American Academy of Anti-Aging Medicine (2014). Available at: https://books.google.com.au/books?hl=en&lr=&id=JEg9BAAAQBAJ&oi=fnd&pg=PP35&dq=Electrosmog+and+Electrosensitivity:+What+Doctors+Need+to+Know+to+Help+their+Patients+Heal (accessed November 29, 2022).

[41] Lamech F. Self-reporting of symptom development from exposure to radiofrequency fields of wireless smart meters in Victoria, Australia: a case series. Altern Ther Health Med. (2014) 20:28–39. 25478801

[42] Stein Y Udasin IG. Electromagnetic hypersensitivity (EHS, microwave syndrome)–review of mechanisms. Environ Res. (2020) 186:109445. 10.1016/j.envres.2020.109445 32289567

[43] World Health Organization. International Classification of Diseases (ICD-10). Geneva: World Health Organization (2019). Available online at: https://icd.who.int/browse10/2019/en#/ (accessed November 29, 2022).

[44] World Health Organization. Electromagnetic Fields and Public Health: Electromagnetic Hypersensitivity. Fact sheet N (2013). Available online at: https://www.who.int/teams/environment-climate-change-and-health/radiation-and-health/non-ionizing/electromagnetic-hypersensitivity pdf (accessed October 29, 2022).

[45] Johansson O. Electrohypersensitivity: a functional impairment due to an inaccessible environment. Rev Environ Health. (2015) 30:311–21. 10.1515/reveh-2015-0018 26613327

[46] Frey AH. Human auditory system response to modulated electromagnetic energy. J Appl Physiol. (1962) 17:689–92. 10.1152/jappl.1962.17.4.689 13895081

[47] Frey AH. Headaches from cellular telephones: are they real and what are the implications? Environ Health Perspect. (1998) 106:101–3. 10.1289/ehp.98106101 9441959 PMC1533043

[48] Nizhelska O Marynchenko L Piasetskyi V. Biological risks of using non- thermal non-ionizing electromagnetic fields. Innov Biosyst Bioeng. (2020) 4:95–109. 10.20535/ibb.2020.4.2.202452

[49] Hillert L Berglind N Arnetz BB Bellander T. Prevalence of self-reported hypersensitivity to electric or magnetic fields in a population-based questionnaire survey. Scand J Work Environ Health. (2002) 28:33–41. 10.5271/sjweh.644 11871850

[50] Hocking B. Preliminary report: symptoms associated with mobile phone use. Occup Med. (1998) 48:357–60. 10.1093/occmed/48.6.357 10024730

[51] Hocking B Westerman R. Neurological abnormalities associated with mobile phone use. Occup Med. (2000) 50:366–8. 10.1093/occmed/50.5.366 10975136

[52] Heuser G Heuser SA. Functional brain MRI in patients complaining of electrohypersensitivity after long term exposure to electromagnetic fields. Rev Environ Health. (2017) 32:291–9. 10.1515/reveh-2017-0014 28678737

[53] Irigaray P Caccamo D Belpomme D. Oxidative stress in electrohypersensitivity self-reporting patients: Results of a prospective *in vivo* investigation with comprehensive molecular analysis. Int J Mol Med. (2018) 42:1885–98. 10.3892/ijmm.2018.3774 30015864 PMC6108880

[54] Belpomme D Campagnac C Irigaray P. Reliable disease biomarkers characterizing and identifying electrohypersensitivity and multiple chemical sensitivity as two etiopathogenic aspects of a unique pathological disorder. Rev Environ Health. (2015) 30:251–71. 10.1515/reveh-2015-0027 26613326

[55] Dieudonné M. Does electromagnetic hypersensitivity originate from nocebo responses? Indications from a qualitative study. Bioelectromagnetics. (2016) 37:14–24. 10.1002/bem.21937 26369906

[56] Redmayne M Reddel S. Redefining electrosensitivity: a new literature- supported model. Electromagn Biol Med. (2021) 40:227–35. 10.1080/15368378.2021.1874971 33492997

[57] Leszczynski D Helsingin Y. The lack of international and national health policies to protect persons with self-declared electromagnetic hypersensitivity. Rev Environ Health. (2022). 10.1515/reveh-2022-0108 36288575

[58] Simkó M Mattsson M-O. 5G wireless communication and health effects—a pragmatic review based on available studies regarding 6 to 100 GHz. Int J Environ Res Public Health. (2019) 16:3406. 10.3390/ijerph16183406 31540320 PMC6765906

[59] Karipidis K Mate R Urban D Tinker R Wood A. 5G mobile networks and health—a state-of-the-science review of the research into low-level RF fields above 6 GHz. J Expo Sci Environ Epidemiol. (2021) 31:585–605. 10.1038/s41370-021-00297-6 33727687 PMC8263336

[60] Wood A Mate R Karipidis K. Meta-analysis of *in vitro* and *in vivo* studies of the biological effects of low-level millimetre waves. J Expo Sci Environ Epidemiol. (2021) 31:606–13. 10.1038/s41370-021-00307-7 33727686 PMC7962924

[61] Weller S May M McCredden J Leach V Phung D Belyaev I. Comment on “5G mobile networks and health-a state-of-the-science review of the research into low-level RF fields above 6 GHz” by Karipidis et al. J Exp Sci Environ Epidemiol. (2022) 1–4. 10.1038/s41370-022-00497-8 36434135 PMC9849131

[62] Betzalel N Ishai PB Feldman Y. The human skin as a sub-THz receiver–Does 5G pose a danger to it or not? Environ Res. (2018) 163:208–16. 10.1016/j.envres.2018.01.032 29459303

[63] Feldman Y Puzenko A Ben Ishai P Caduff A Davidovich I Sakran F. The electromagnetic response of human skin in the millimetre and submillimetre wave range. Phys Med Biol. (2009) 54:3341. 10.1088/0031-9155/54/11/005 19430110

[64] Neufeld E Kuster N. Systematic derivation of safety limits for time-varying 5G radiofrequency exposure based on analytical models and thermal dose. Health Phys. (2018) 115:705–11. 10.1097/HP.0000000000000930 30247338

[65] Zalyubovskaya NP. Biological effects of millimeter radiowaves. Vrachebnoye Delo. (1977) 3:116–9. Available online at : https://stopsmartmetersau.files.wordpress.com/2019/03/biological-effect-of-millimeter-radiowaves.pdf (accessed November 26, 2022).

[66] Ziskin MC. Millimeter waves: acoustic and electromagnetic. Bioelectromagnetics. (2013) 34:3–14. 10.1002/bem.21750 22926874 PMC3522782

[67] Usichenko TI Edinger H Gizhko VV Lehmann C Wendt M Feyerherd F. Low-intensity electromagnetic millimeter waves for pain therapy. Evid Based Complement Alternat Med. (2006) 3:201–7. 10.1093/ecam/nel012 16786049 PMC1475937

[68] Leszczynski D. Physiological effects of millimeter-waves on skin and skin cells: an overview of the to-date published studies. Rev Environ Health. (2020) 35:493–515. 10.1515/reveh-2020-0056 32829319

[69] International Commission on Non-Ionizing Radiation Protection. Guidelines for limiting exposure to electromagnetic fields (100 kHz to 300 GHz). Health Phys. (2020) 118:483–524. 10.1097/HP.0000000000001210 32167495

[70] Tanlaka EF Ewashen C King-Shier K. Postpositivist critical multiplism: its value for nursing research. Nursing open. (2019) 6:740–4. 10.1002/nop2.306 31367395 PMC6650753

[71] Becker RO. Cross Currents: The Perils of Electropollution, The Promise of Electromedicine., ed Jeremy P. New York, NY: Tarcher / Penguin (1990).

[72] Belyaev I. Dependence of Non-Thermal Biological Effects of Microwaves on Physical Biological Variables: Implications for Reproducibility Safety Standards. Non- thermal effects mechanisms of interaction between electromagnetic fields living matter. Bologna (IT): Ramazzini Institute (2010). p. 187–218. Available online at: https://www.researchgate.net/publication/284970330 (accessed October 29, 2022).

[73] Fröhlich H. Biological Coherence and Response to External Stimuli. (2012). Springer Science and Business Media.

[74] Adey WR. Biological effects of electromagnetic fields. J Cell Biochem. (1993) 51:410–6. 10.1002/jcb.2400510405 8388394

[75] Panagopoulos DJ Messini N Karabarbounis A Philippetis AL Margaritis LHl. A mechanism for action of oscillating electric fields on cells. Biochem Biophys Res Commun. (2000) 272:634–40. 10.1006/bbrc.2000.2746 10860806

[76] Panagopoulos DJ Karabarbounis A Margaritis LH. Mechanism for action of electromagnetic fields on cells. Biochem Biophys Res Commun. (2002) 298:95–102. 10.1016/S0006-291X(02)02393-8 12379225

[77] Pall ML. Electromagnetic fields act via activation of voltage-gated calcium channels to produce beneficial or adverse effects. J Cell Mol Med. (2013) 17:958–65. 10.1111/jcmm.12088 23802593 PMC3780531

[78] Pall ML. Wi-Fi is an important threat to human health. Environ Res. (2018) 164:405–16. 10.1016/j.envres.2018.01.035 29573716

[79] Calabrò E Magazù S. Parallel β-sheet vibration band increases with proteins dipole moment under exposure to 1765 MHz microwaves. Bioelectromagnetics. (2016) 37:99–107. 10.1002/bem.21956 26833949

[80] Blank M Goodman R. Electromagnetic fields stress living cells. Pathophysiology. (2009) 16:71–8. 10.1016/j.pathophys.2009.01.006 19268550

[81] Carlo GL. Radio waves, wireless signals, and public health: is this the next silent spring? Environ Claims J. (2000) 12:55–77. 10.1080/10406020009355139

[82] Bandara P Weller S. Cardiovascular disease: time to identify emerging environmental risk factors. Eur J Prev Cardiol. (2017) 24:1819–23. 10.1177/2047487317734898 28969497

[83] Belyaev I Markov MS. Biophysical mechanisms for nonthermal microwave effects. In:Markov MS, editor. Electromagnetic Fields in Biology and Medicine. Boca Raton, FL: CRC Press (2015). p. 49–68. 10.1201/b22486

[84] Hinrikus H Bachmann M Lass J. Understanding physical mechanism of low- level microwave radiation effect. Int J Radiat Biol. (2018) 94:877–82. 10.1080/09553002.2018.1478158 29775391

[85] Giuliani L Soffritti M. Non-Thermal Effects and Mechanisms of Interaction Between Electromagnetic Fields and Living Matter. Bologna, Italy: National Institute for the Study and Control of Cancer and Environmental Diseases “Bernardino Ramazzini” (2010).

[86] Hinrikus H Karpowicz J Naarala J. Special issue: electromagnetic fields in biology and medicine. Int J Radiat Biol. (2018) 94:873–6. 10.1080/09553002.2018.1533359 30321099

[87] Frey AH. Cardiac and neural effects of modulated RF energy. In: Proceedings of the 23rd Annual Conference on Engineering in Medicine and Biology. Washington, DC (1970).

[88] ICRP. The 2007 recommendations of the international commission on radiological protection. ICRP publication 103, in Annals of the ICRP, International Commission on Radiological Protection. (2007). Available online at: https://www.icrp.org/publication.asp?id=ICRP%20Publication%20103 (accessed October 29, 2022).

[89] The Resolution of Thematic issue on the materials of the 3rd International Forum of the Scientific Council of the Russian Federation on human ecology and environmental hygiene on the topic: “Modern problems of assessing forecasting and managing environmental risks to public health and the environment, ways for their rational solution”. Hyg Sanit. (2019) 98:1321–2. (In Russ.) Available online at: https://www.rjhas.ru/jour/article/view/459?locale=en_US (accessed November 29, 2022).

[90] International Commission on the Biological Effects of Electromagnetic Fields (ICBE- EMF). Scientific evidence invalidates health assumptions underlying the FCC and ICNIRP exposure limit determinations for radiofrequency radiation: implications for 5G. Environ Health. (2022) 21:92. 10.1186/s12940-022-00900-9 36253855 PMC9576312

[91] Hardell L. World Health Organization, radiofrequency radiation and health-a hard nut to crack. Int J Oncol. (2017) 51:405–13. 10.3892/ijo.2017.4046 28656257 PMC5504984

[92] Nordhagen EK Flydal E. Self-referencing authorships behind the ICNIRP 2020 radiation protection guidelines. Rev on Environ Health. (2022). 10.1515/reveh-2022-0037 35751553

[93] Kane RC. Cellular Telephone Russian Roulette: A Historical Scientific Perspective. New York, NY: Vantage Press (2001). Available online at: https://www.icems.eu/docs/Robert_C_Kane.pdf (accessed November 29, 2022).

[94] Leach V Bromwich D. Why the Precautionary Approach is Needed for Non-Ionising Radiation Devices. Radiation Protection in Australasia (2018). Available online at: https://www.orsaa.org/uploads/6/7/7/9/67791943/why_the_precautionary_approach_is_needed_for_non-ionising_radiation_devicesarps_journal_vol_35_no_1_pages_13_to_21.pdf (accessed October 29, 2022).

[95] Weller S Leach V May M. Comment on letter: “Post-normal science and the management of uncertainty in bioelectromagnetic controversies” by AW Wood. Bioelectromagnetics. (2020) 41:80–4. 10.1002/bem.22225 31608459

[96] Gee D. Late lessons from early warnings: towards realism and precaution with EMF? Pathophysiology. (2009) 16:217–31. 10.1016/j.pathophys.2009.01.004 19467848

[97] Carpenter DO Sage C. Setting prudent public health policy for electromagnetic field exposures. Rev Environ Health. (2008) 23:91–118. 10.1515/REVEH.2008.23.2.91 18763539

[98] Dämvik M Johansson O. Health risk assessment of electromagnetic fields: a conflict between the precautionary principle and environmental medicine methodology. Rev Environ Health. (2010) 25:325–34. 10.1515/REVEH.2010.25.4.325 21268445

[99] American Nurses Association. Code of Ethics for Nurses With Interpretive Statements. Silver Spring, MD: American Nurses Association, Nursebooks.org (2001). Available online at: https://www.nursingworld.org/practice-policy/nursing-excellence/ethics/code-of-ethics-for-nurses/coe-view-only/ (accessed November 29, 2022).

[100] Havas M Marrongelle J Pollner B Kelley E Rees CR Tully L. Provocation study using heart rate variability shows microwave radiation from 2.4 GHz cordless phone affects autonomic nervous system. Eur J Oncol Library. (2010) 5:273–300. Available online at: https://www.researchgate.net/publication/228993615 (accessed November 26, 2022).

[101] Peel J. When (scientific) rationality rules: (mis) application of the precautionary principle in Australian mobile phone tower cases: Telstra corporation limited v Hornsby shire Council. J Environ Law. (2007) 19:103–20. 10.1093/jel/eqm002

[102] Physicians' health initiative for radiation and environment. Radiofrequency Radiation Reduction How To? 2021. Available online at: https://phiremedical.org/resources/radiofrequency-radiation-reduction-how-to/. (accessed October 29, 2022).

[103] Physicians' health initiative for radiation and environment. 2020 Consensus Statement of UK and International Medical and Scientific Experts and Practitioners on Health Effects of Non-Ionising Radiation (NIR). (2021). Available online at: https://phiremedical.org/2020-nir-consensus-statement-read/ (accessed October 29, 2022).

[104] Davis D. What Cell Phones Do To Your Bod, TEDx Talk. (2020). Available online at: https://ehtrust.org/ (accessed October 29, 2022).

[105] Carducci AL Agodi A Ancona C Angelini P Bagordo F Barbone F. Impact of the environment on the health: From theory to practice. Environ Res. (2021) 194:110517. 10.1016/j.envres.2020.110517 33271142

[106] Chandel RS Sharma S Kaur S Singh S Kumar R. Smart watches: a review of evolution in bio-medical sector. Mater Today Proc. (2021) 50:1053–66. 10.1016/j.matpr.2021.07.460

