# Supplementary material for: Wireless technology is an environmental stressor requiring new understanding and approaches in health care
Source: Front Public Health. 2022 Dec 20;10:986315. doi: 10.3389/fpubh.2022.986315 (PMC9809975; doi:10.3389/fpubh.2022.986315)
Supplement: Supplementary file 2 [file Data_Sheet_2.pdf]

Supplement 2: Summary tables showing categorised papers produced by three ODEB search queries for (i) Experimental (*in vitro and in vivo*) studies, (ii) Epidemiological studies, and (iii) Experimental (*in vitro and in vivo*) studies where real mobile phone and WiFi signals were used in experiments.

(i) Effects categories summary from ODEB June 2020
https://www.orsaa.org/orsaa-database.html
Selected UHF frequencies (300 kHz to 300 GHz)
In layout APAD (file/ manage layouts / APAD)
Experimental (*in vitro and in vivo*) studies.

Find Search Summary Totals

Peer Reviewed Studies Showing Biological Effects Number of records used : 1213 of 4468

Auditory Dysfunction / Hearing loss / Tinnitus	7	Apoptosis (Programmed Cell Death)	107	Brain Tumours	0
Blood Brain Barrier Permeability Changes	12	Breast Cancer	1	Cellular Stress	56
Brain Development / Neuro Degeneration	52	Biochemical Changes	351	EEG changes / Brain Waves	24
Neuro Behavioural Effect / Cognitive Effects	85	Cell Irregularities/ Damage/ Morphological Changes	206	Effects on Mitochondria	45
Calcium Influx / Efflux	15	Fatigue	2	Altered Enzyme Activity / Protein Levels / Protein Damage	340
Circadian Rhythm Disruption	8	Altered Gene Expression	158	Headaches/Migraines	2
DNA Damage / Mutagenic / Genotoxic	169	Altered Glucose Level / Glucose Metabolism	16	Inflammation	19
Endocrine / Hormone Effects	72	Cardiovascular Effects	28	Hepatic Effects (Liver)	37
Miscarriage / Spontaneous Abortion / Foetus Resorption	1	Immune System Effects	54	Impaired / Reduced Healing/ Bone Density Changes	6
Memory Impairment	34	Oxidative Stress / ROS/ Free Radicals	239	Speech Impairment	0
Sperm /Testicular Effects	97	Sleep Effects	8	Haematological Effects	75
Tumour Promotion	12	Neurotransmitter Effects	30	Synergistic/Combinative Effects	60
Thyroid Effects	20	Visual Disturbances/ Ocular Effects	19	Autism	4
Leukemia	0	Parotid Gland Malignancy	0	Neoplasis/ Hyperplasia (Abnormal Tissue Growth)	6
Depression	0	Induced Adaptive Response	42	Dizziness / Vertigo / Vestibular Effects	0

May have a role in disease pathway/ well-being A known disease Continue

(ii) Effects categories summary from ODEB June 2020
https://www.orsaa.org/orsaa-database.html
Selected UHF frequencies (300 kHz to 300 GHz)
In layout APAD (file/ manage layouts / APAD)
Epidemiological studies.

Find Search Summary Totals

Peer Reviewed Studies Showing Biological Effects — Number of records used : 251 of 4468

Auditory Dysfunction / Hearing loss / Tinnitus	14	Apoptosis (Programmed Cell Death)	0	Brain Tumours	17
Blood Brain Barrier Permeability Changes	0	Breast Cancer	3	Cellular Stress	1
Brain Development / Neuro Degeneration	1	Biochemical Changes	27	EEG changes / Brain Waves	0
Neuro Behavioural Effect / Cognitive Effects	45	Cell Irregularities/ Damage/ Morphological Changes	14	Effects on Mitochondria	0
Calcium Influx / Efflux	0	Fatigue	32	Altered Enzyme Activity / Protein Levels / Protein Damage	11
Circadian Rhythm Disruption	2	Altered Gene Expression	1	Headaches/Migraines	49
DNA Damage / Mutagenic / Genotoxic	27	Altered Glucose Level / Glucose Metabolism	1	Inflammation	3
Endocrine / Hormone Effects	19	Cardiovascular Effects	19	Hepatic Effects (Liver)	2
Miscarriage / Spontaneous Abortion / Foetus Resorption	3	Immune System Effects	15	Impaired / Reduced Healing/ Bone Density Changes	2
Memory Impairment	19	Oxidative Stress / ROS/ Free Radicals	9	Speech Impairment	3
Sperm /Testicular Effects	26	Sleep Effects	35	Haematological Effects	14
Tumour Promotion	10	Neurotransmitter Effects	5	Synergistic/Combinative Effects	5
Thyroid Effects	8	Visual Disturbances/ Ocular Effects	17	Autism	2
Leukemia	4	Parotid Gland Malignancy	3	Neoplasis/ Hyperplasia (Abnormal Tissue Growth)	3
Depression	17	Induced Adaptive Response	0	Dizziness / Vertigo / Vestibular Effects	17

May have a role in disease pathway/ well-being — A known disease — Continue

(iii) Effects categories summary from ODEB June 2020
https://www.orsaa.org/orsaa-database.html
Selected UHF frequencies (300 kHz to 300 GHz)
In layout APAD (file/ manage layouts / APAD)
Experimental (*in vitro and in vivo*) studies
Real mobile phones and WiFi signals were used in experiments.

Find Search Summary Totals

Peer Reviewed Studies Showing Biological Effects Number of records used : **323** of **4468**

Auditory Dysfunction / Hearing loss / Tinnitus	1	Apoptosis (Programmed Cell Death)	24	Brain Tumours	0
Blood Brain Barrier Permeability Changes	4	Breast Cancer	1	Cellular Stress	10
Brain Development / Neuro Degeneration	19	Biochemical Changes	100	EEG changes / Brain Waves	1
Neuro Behavioural Effect / Cognitive Effects	33	Cell Irregularities/ Damage/ Morphological Changes	59	Effects on Mitochondria	4
Calcium Influx / Efflux	2	Fatigue	2	Altered Enzyme Activity / Protein Levels / Protein Damage	102
Circadian Rhythm Disruption	2	Altered Gene Expression	29	Headaches/Migraines	2
DNA Damage / Mutagenic / Genotoxic	38	Altered Glucose Level / Glucose Metabolism	7	Inflammation	6
Endocrine / Hormone Effects	20	Cardiovascular Effects	11	Hepatic Effects (Liver)	11
Miscarriage / Spontaneous Abortion / Foetus Resorption	0	Immune System Effects	21	Impaired / Reduced Healing/ Bone Density Changes	2
Memory Impairment	11	Oxidative Stress / ROS/ Free Radicals	79	Speech Impairment	0
Sperm /Testicular Effects	40	Sleep Effects	1	Haematological Effects	27
Tumour Promotion	3	Neurotransmitter Effects	6	Synergistic/Combinative Effects	10
Thyroid Effects	9	Visual Disturbances/ Ocular Effects	4	Autism	1
Leukemia	0	Parotid Gland Malignancy	0	Neoplasis/ Hyperplasia (Abnormal Tissue Growth)	0
Depression	0	Induced Adaptive Response	7	Dizziness / Vertigo / Vestibular Effects	0

May have a role in disease pathway/ well-being   A known disease   **Continue**
