# Supplementary material for: Wireless technology is an environmental stressor requiring new understanding and approaches in health care
Source: Front Public Health. 2022 Dec 20;10:986315. doi: 10.3389/fpubh.2022.986315 (PMC9809975; doi:10.3389/fpubh.2022.986315)
Supplement: Supplementary file 3 [file Data_Sheet_3.pdf]

Supplement 3: PRISMA-like flow diagram for selecting experimental papers using real-world signals

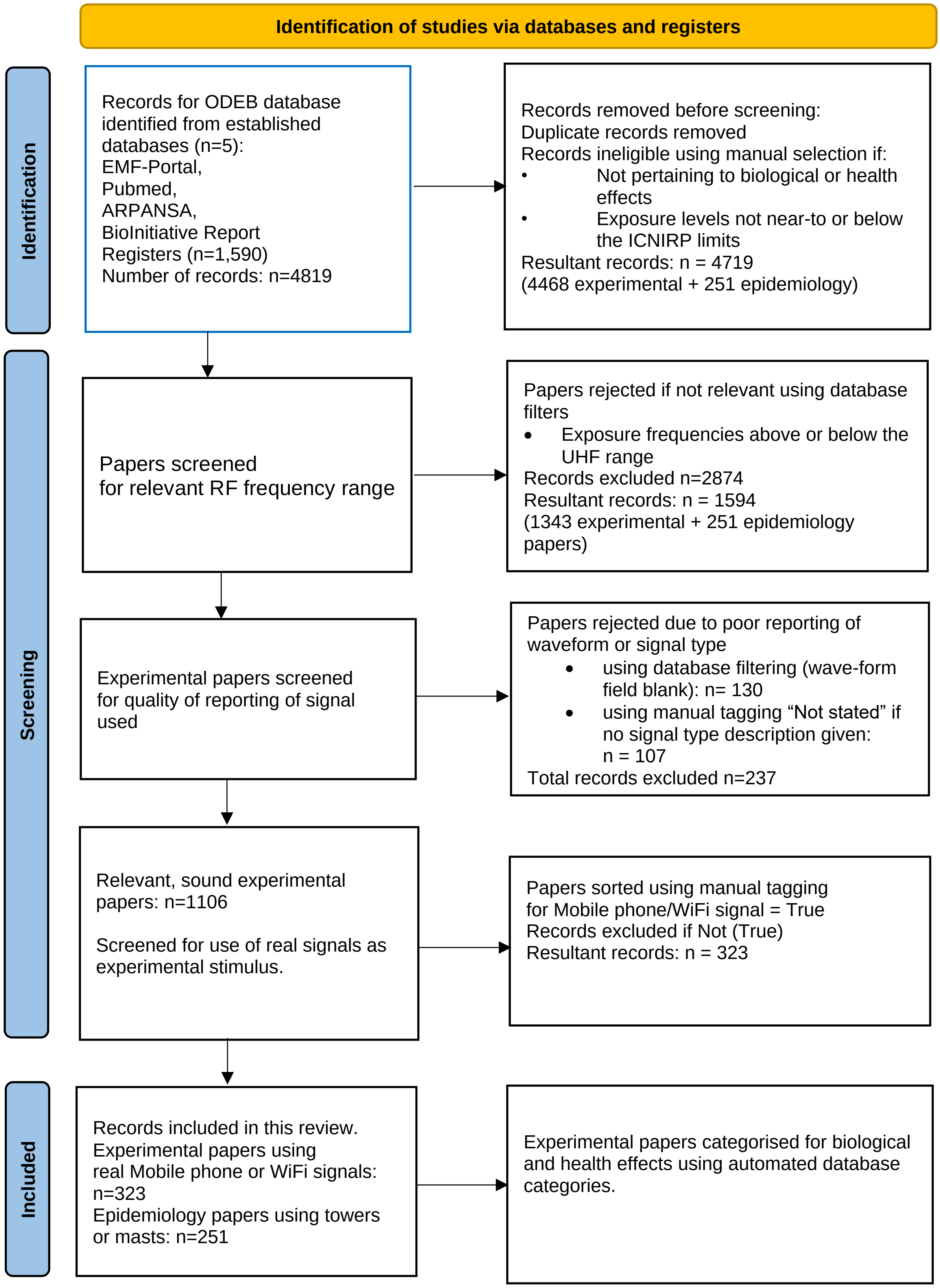

Based on guidelines in: Page MJ, McKenzie JE, Bossuyt PM, Boutron I, Hoffmann TC, Mulrow CD, et al. The PRISMA 2020 statement: an updated guideline for reporting systematic reviews. BMJ 2021;372:n71. doi: 10.1136/bmj.n71

For more information, visit: http://www.prisma-statement.org/
